# Waist height ratio predicts chronic kidney disease: a systematic review and meta-analysis, 1998–2019

**DOI:** 10.1186/s13690-019-0379-4

**Published:** 2019-12-18

**Authors:** Ling Liu, Yanqiu Wang, Wanjun Zhang, Weiwei Chang, Yuelong Jin, Yingshui Yao

**Affiliations:** School of Public Health,Wannan Medical College, Wenchang West Road 22, Wuhu, China

**Keywords:** Chronic kidney disease, Obesity, Physical measurement index, Waist-to-height ratio

## Abstract

**Background:**

The incidence of chronic kidney disease (CKD) increases each year, and obesity is an important risk factor for CKD. The main anthropometric indicators currently reflecting obesity are body mass index (BMI), waist circumference (WC), waist-to-hip ratio (WHR) and waist-to-height ratio (WHtR), but the rationality and merits of various indicators vary. This article aims to find whether the WHtR is a more suitable physical measurement that can predict CKD.

**Methods:**

Pubmed, embase, the cochrane library, and web of science were systematically searched for articles published between 1998 and 2019 screening CKD through physical indicators. Two reviewers independently screened the literature according to the inclusion and exclusion criteria, extracted the data, and evaluated the quality of the methodology included in the study. Meta-analysis used the Stata 12.0 software.

**Results:**

Nine studies were included, with a total of 202,283 subjects. Meta-analysis showed that according to the analysis of different genders in 6 studies, regardless of sex, WHtR was the area with the largest area under the curve (AUC). Except WHtR and visceral fat index (VFI) in women which showed no statistical difference, WHtR and other indicators were statistically different. In three studies without gender-based stratification, the area under the curve AUC for WHtR remained the largest, but only the difference between WHtR and BMI was statistically significant. When the Chinese population was considered as a subgroup, the area under the curve AUC for WHtR was the largest. Except for WHtR and VFI which showed no statistical difference in women, there was a statistically significant difference between WHtR and other indicators in men and women.

**Conclusion:**

WHtR could be better prediction for CKD relative to other physical measurements. It also requires higher-quality prospective studies to verify the clinical application of WHtR.

## Background

Chronic kidney disease (CKD) is a relatively common disease with a prevalence of approximately 10% in the United States, 7% in the United Kingdom, 13% in Japan, and 10.8% in China. It not only increases the risk of end-stage renal disease (ESRD), but also increases the probability of cardiovascular disease and premature death [1–4]. Diabetes, chronic nephritis, hypertension, and obesity are risk factors for chronic kidney disease [5]. Dialysis and kidney transplantation are the main alternatives to end-stage renal disease, but both of these treatment methods have a huge economic burden on patients, their families, and society. Their complications and outcomes, while reducing the quality of life and life expectancy, increase the economic burden on individuals and society. Therefore, screening for CKD is of great significance.

Obesity, as an increasingly common metabolic disease, has attracted widespread attention due to its co-occurance with metabolic-related diseases such as induced hypertension, hyperlipidemia, and diabetes. These diseases are also related to the occurrence of CKD. A 2009 population census showed that two-thirds of adult Americans have excess body mass, and half of them are obese [6].

At present, the main anthropometric indicators that reflect obesity are body mass index (BMI), waist circumference (WC), waist-to-hip ratio (WHR), and waist circumference to height ratio (WHtR). Compared with subcutaneous fat, visceral fat has higher metabolic and inflammatory activity and is more closely linked to renal damage [7]. Since BMI is an index for assessing general body fat, it cannot differentiate between fat, muscle, and edema or between visceral fat and subcutaneous fat. Although some studies have shown a clear correlation between BMI and CKD not all studies are consistent [8]. Ashwell et al. [9] examined, BMI, WC, and WHtR, commonly used obesity indicators, in a meta-analysis of epidemiological surveys and found that WHtR is superior to BMI and WC in assessing cardiovascular risk. Previous work by Elsayed et al. 2008 found an association between WHR and CKD [10]. However, few studies have shown a link between WHtR and CKD. Thus, we hypothesize that the abdominal obesity index will predict CKD more accurately than BMI.

To investigate whether WHtR can better reflect the influence of obesity on chronic kidney disease, we conducted a systematic meta-analysis to evaluate the predictive value of WHtR on CKD and provide scientific basis for effective prevention of CKD.

## Data and methods

### Search strategy

We searched pubmed, embase, the cochrane library, and the web of science database for articles published from 1998 to 2019. The search was performed using the keywords: waist height ratio, waist-stature ratio, chronic kidney disease, chronic renal disease, and receiver operating characteristic curve (ROC). Retrospective study were also included, and if necessary, the author was contacted for relevant information.

### Inclusion and exclusion criteria

#### Inclusion criteria

(1) All subjects included in the study were 18 years of age or older. (2) Outcome measures included chronic kidney disease and the diagnostic criteria of an estimated glomerular filtration rate (eGFR) < 60 ml/min/1.73m^2^. (3) The CKD risk factors studied included obesity or being overweight and degree of obesity was measured using WHtR and WC or WHR or BMI. (4) The results of the study reported the area under the curve (AUC) and the confidence interval (CI) of the AUC for the CKD test and the efficacy of the measures used. (5) The study type was a cross-sectional study or cohort study. (6) Languages were limited to Chinese and English.

#### Exclusion criteria

(1) The results did not contain the AUC of obesity indicators such as WHtR and BMI used to predict CKD. (2) The reported AUC gender is unclear or unspecified whether it is stratified by gender. (3) Logical error in the extracted data or inappropriate analysis method. (4) Duplicate publication or use of the data has been published only from more comprehensive reports.

### Literature screening and quality evaluation

Two researchers strictly selected literature, extracted data, and assessed quality based on the inclusion and exclusion, and finally cross-checked. In the event of disagreement, it was discussed or submitted to a third person to assist in adjudication.

The main information for data extraction includes: (1) The author of the document, when the study was conducted, and the country or region. (2) Study design type and data source. (3) Sample size, age, and sex distribution of the study subjects. (4) CKD prevalence, value of each obesity measure, and AUC with 95% CI for CKD.

Quality assessment of cohort studies and case-control studies was performed using the Newcastle-Ottawa Scale (NOS) quality assessment tool. Assessment of cross-sectional studies was performed using the Agency for Healthcare Research and Quality (AHRQ) assessment tool. The rating is divided into “yes” if the study satisfies the criterea, “no” if it does not meet the criteria, and “unclear” if the quality could not be determined.

### Statistical methods

Cochrane χ^2^ test was used to judge whether there was heterogeneity among the included articles, and the *I*^2^ statistic was used to quantify the degree of heterogeneity. According to the results of the heterogeneity test, an appropriate effect-value combination model was selected. If the heterogeneity test was *I*^2^ ≤ 50% and *P* ≥ 0.10, a fixed effect model was used for analysis. If the heterogeneity test was *I*^2^ > 50%, *P* < 0.10, a random effect model was used for analysis. Student’s t-test was used for single-sample or two-sample measurements, analysis of variance was used for multiple samples, chi-square test was used for counts. The reciprocal method of variance was used to obtain the combined effect. Whether or not the 95% CI of the combined values overlap was used to determine if there is a difference between the different combined values. If they overlap, they cannot be considered statistically significant, if they do not overlap then they are statistically different. Sensitivity analysis was performed on all included studies and publication bias was assessed using the Egger regression asymmetry test. The above analysis was performed using Stata 12.0 software.

## Results

### Literature screening results

**Fig. 1:**
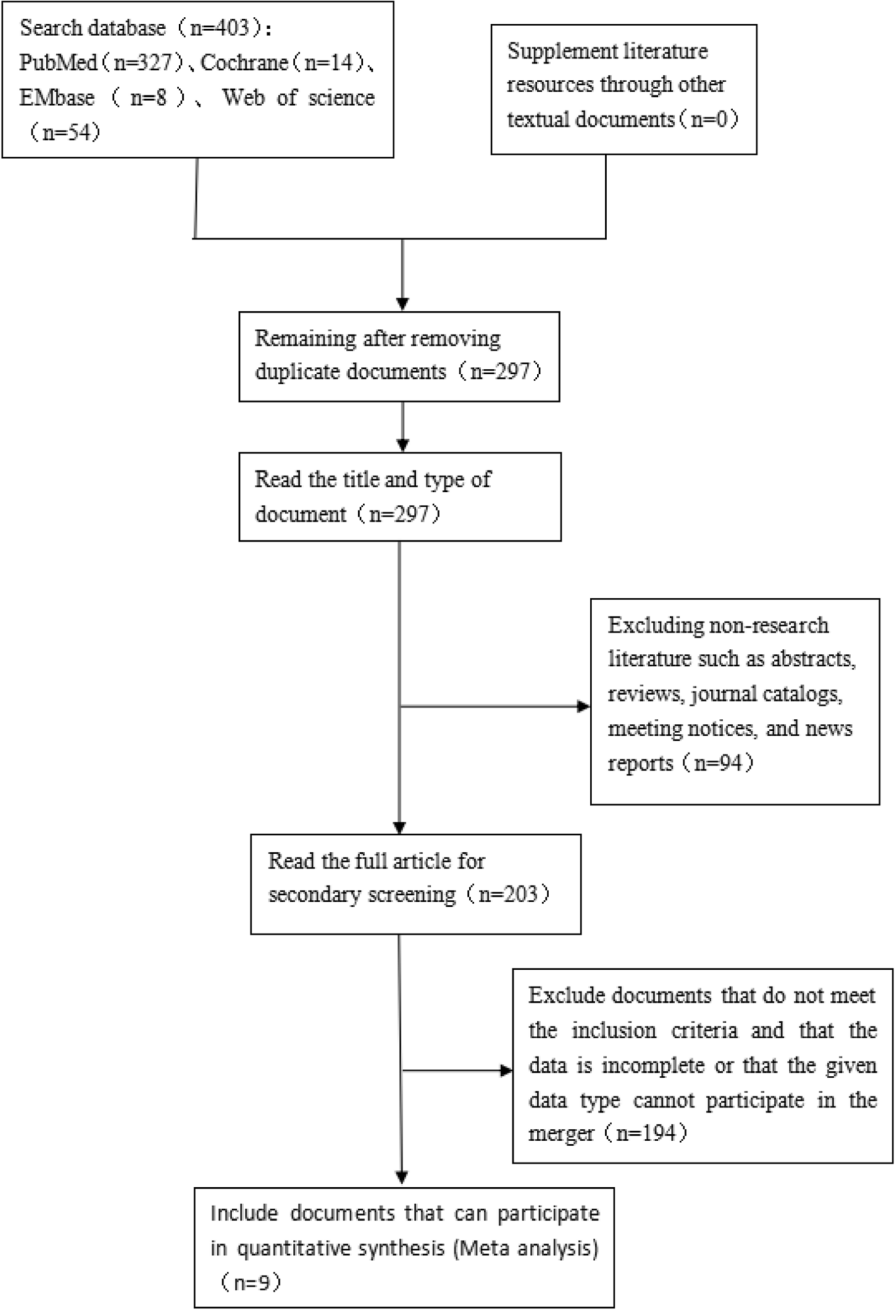
Document screening process. A systematic review and meta-analysis on the waist height and chronic kidney disease, 1998-2019 [32]

A total of 403 related articles were retrieved, and 9 studies were eventually included according to the inclusion and exclusion criteria (Fig. 1).

### Main characteristics and quality assessment of the study

The study was conducted between 2003 and 2015 in east Asia and Europe. Four of the study populations were from representative populations in the study site, and the other five were physical examinations at hospitals or physical examination centers. A total of 202,283 people, 119,214 males and 83,069 females, with 201,428 from east Asia and 855 from Europe were enrolled. The ages of the population were mostly concentrated between 35 and 70 years old. There were 8 cross-sectional studies and 1 cohort study included (Table 1).

**Table 1:** The main characteristics and quality evaluation of the included studies (by gender). A systematic review and meta-analysis on the waist height and chronic kidney disease, 1998–2019

Inclusion study	Research area	Research period	Research methods	Data Sources	Sample size (n)		Age		Number of CKD patients (n)		Quality Evaluation
					male	female	male	female	male	female	
Dong 2018 [11]	China	2012–2015	cross-sectional survey	Randomly sampled population survey data across 31 provinces and cities in the Middle East and West China	13,410	16,106	56.48 ± 13.13	56.48 ± 13.13	683	895	8
Dai 2016 [12]	China	2012–2013	cross-sectional survey	Multi-stage stratified randomized cluster sampling data from representative populations in Liaoning Province, China	5168	6024	≥35	≥35	85	152	8
Jaroszynski 2016 [13]	Britain	–	cross-sectional survey	Population survey data for some rural areas in the United Kingdom	–	730	–	71.4 ± 4.98	–	89	8
Odagiri 2014 [14]	Japan	2008–2011	cohort study	Japanese company employees’ medical data	3686	1155	18–67	18–67	300	84	7
Liu 2016 [15]	China	2013–2014	cross-sectional survey	Hospital health checkup data in Hunan Province	15,593	11,062	18–80	18–80	1496	338	9
He 2016 [16]	China	2008–2009	cross-sectional survey	Multi-stage cluster sampling health check data for Chinese cities	78,142	45,487	45.1 ± 14.2	44.3 ± 13.5	4629	1516	8
Bulum 2016 [17]	Croatia	–	cross-sectional survey	The annual physical examination data of T2DM consecutive male and female patients	65	60	31–76		36		9
Lin 2007 [18]	China	2003–2005	cross-sectional survey	Taizhou City University of Traditional Chinese Medicine Affiliated Hospital Crowd Health Examination Data	2613	1998	28–83		221		9
Chou 2008 [19]	China	2003–2005	cross-sectional survey	Population health check data at Affiliated Hospital of China Medical University	537	447	66.7 ± 5.3		161		8

Nine studies were evaluated for quality based on NOS and AHRQ literature quality assessment criteria. All included articles provided information on most of the evaluation items and study quality was generally high (Table 1). A summary of obesity indicators used in each study is shown in Table 2.

**Table 2:** Anthropometric baseline data included in the study. A systematic review and meta-analysis on the waist height and chronic kidney disease, 1998–2019

Inclusion study	BMI (kg/m^2^)		WC (cm)		PBF		VFI		WHtR		WHR	
	male	female	male	female	male	female	male	female	male	female	male	female
Dong 2018 [11]	24.28 ± 3.64	24.80 ± 4.03	85.73 ± 10.77	84.75 ± 11.34	26.59 ± 5.51	35.16 ± 5.78	12.02 ± 5.59	9.12 ± 4.86	0.52 ± 0.06	0.56 ± 0.07	–	–
Dai 2016 [12]	24.73 ± 3.55	24.85 ± 3.75	83.77 ± 9.74	81.23 ± 9.70	–	–	1.73 ± 2.20	2.36 ± 2.40	0.50 ± 0.06	0.52 ± 0.06	–	–
Jaroszynski 2016 [13]	–	30.64 ± 5.26	–	98.02 ± 12.50	–	–	–	–	–	0.623 ± 0.082	–	0.870 ± 0.085
Odagiri 2014 [14]	–	–	–	–	–	–	–	–	–	–	–	–
Liu 2016 [15]	25.49 ± 3.63	23.32 ± 3.39	88.60 ± 9.66	78.69 ± 9.13	–	–	–	–	0.53 ± 0.06	0.50 ± 0.06	–	–
He 2016 [16]	24.8 ± 3.2	22.6 ± 3.3	85.5 ± 9.0	74.3 ± 8.6	–	–	–	–	0.50 ± 0.05	0.47 ± 0.06	–	–
Bulum 2016 [17]	35.57 ± 5.36		119 (88–192)		–	–	–	–	0.674 (0.321–1.091)		1.005 (0.795–1.401)	
Lin 2007 [18]	24.5 ± 3.3	23.1 ± 3.6	86.9 ± 9	77.8 ± 9.5	–	–	–	–	0.52 ± 0.05	0.5 ± 0.06	0.9 ± 0.06	0.82 ± 0.07
Chou 2008 [19]	24.3 ± 3.5		85.9 ± 9.7		–	–	–	–	0.54 ± 0.06		0.89 ± 0.07	

*BMI* Body Mass Index, *WC* Waist Circumference, *PBF* Percentage of Body Fat, *VFI* Visceral Fat Index, *WHtR* Waist Circumference Height Ratio, *WHR* Waist-to-hip Ratio

### Meta-analysis results

#### Stratified research results based on gender analysis

A total of 6 studies were stratified according to gender. The heterogeneity test of BMI, WC, VFI, and WHtR in male and female populations for chronic kidney disease had AUC *I*^2^ above 90%, *P* < 0.10 indicating a large degree of heterogeneity. Therefore, the AUC of CKD in the male and female populations were combined using the random effects model. The analysis results showed that WHtR had the greatest combined value of CKD regardless of sex, and 95% *CI* did not overlap, *P* < 0.05. However, there was no statistical difference between the AUC of VFI and BMI on CKD in women. The combined value of WHtR in men was greater than that in women, *P* < 0.05, and the difference was statistically significant (Table 3).

**Table 3:** AUC and heterogeneity test of body indicators of people stratified by sex. A systematic review and meta-analysis on the waist height and chronic kidney disease, 1998–2019

Obesity index	Male			Female		
	AUC	95%*CI*	*I*^2^ (%)	AUC	95%*CI*	*I*^2^ (%)
BMI	0.557	0.554–0.560	97.4^*^	0.55	0.547–0.554	97.6^*^
WC	0.572	0.568–0.575	98.1^*^	0.574	0.570–0.577	55.7
WHtR	0.595	0.592–0.599	97.8^*^	0.587	0.583–0.591	90.3^*^
VFI	0.55	0.542–0.558	90.1^*^	0.582	0.574–0.589	96.9^*^

*P < 0.01

#### Non-sex stratified population-wide study results

Three studies did not stratify based on gender. The heterogeneity test found that the BMI, WC, WHR, and WHtR of the whole population were below *I*^2^ < 50% for the AUC heterogeneity test of CKD, and *P* > 0.1, so there was no heterogeneity. Therefore, indicators of the whole population were combined using the fixed effect model for the AUC of CKD. This analysis showed that WHtR had the greatest combined value of AUC for CKD, but it was only statistically different from BMI, *P* < 0.05. There were no significant differences from the other two indicators, *P* > 0.05 (Table 4).

**Table 4:** AUC and heterogeneity test of body indicators in populations without gender stratification. A systematic review and meta-analysis on the waist height and chronic kidney disease, 1998–2019

Obesity index	AUC	95%*CI*	*I*^2^ (%)
BMI	0.558	0.537–0.580	27.4
WC	0.58	0.558–0.602	0
WHtR	0.606	0.584–0.627	47.8
WHR	0.602	0.581–0.623	0

*P < 0.01

#### Subgroup analysis of Chinese population

To further examine the data, analysis was performed on a subgroup of 4 studies of Chinese populations stratified by gender. Heterogeneity between studies in the male and female populations was higher than that in the subgroups. In addition to female’s WC, the *I*^2^ for each other index was greater than 50%. Therefore, except for female’s WC use of a fixed-effects model for the AUC of CKD, all other measurements were combined using a random effects model. The results of this analysis show that WHtR is the largest combined value of AUC for CKD regardless of gender. For the same gender, WHtR was higher than the measured values, and the difference was statistically significant, *P* < 0.05, with the exception of women where the difference between WHtR and VFI was not statistically significant, *P* > 0.05 (Table 5).

**Table 5:** Subgroup analysis results of AUC and heterogeneity tests for body measurements in Chinese population. A systematic review and meta-analysis on the waist height and chronic kidney disease, 1998–2019

Obesity index	Male			Female		
	AUC	95%*CI*	*I*^2^ (%)	AUC	95%*CI*	*I*^2^ (%)
BMI	0.557	0.554–0.560	98^*^	0.55	0.546–0.554	98.6^*^
WC	0.571	0.568–0.574	98.6^*^	0.573	0.569–0.577	0
WHtR	0.595	0.592–0.598	98.3^*^	0.586	0.582–0.590	92.5^*^
VFI	0.55	0.542–0.558	90.1^*^	0.582	0.574–0.589	96.9^*^

*P < 0.01

### Sensitivity analysis and publication Bias

To prevent bias in the results of of the study due to low-quality literature, sensitivity analyses were performed on all literature included. The analysis showed that the combined value of WHtR for each gender and the whole population was greater than other obesity measurement indicators, suggesting that the results of this study are consistent.

Egger’s test was used to test the publication bias of each indicator. The results showed that regardless of gender, the *P* values corresponding to each index were greater than 0.05 (Table 6). The results of the funnel plot analysis for Stata 12.0 showed that each circle represented an incorporated study that was approximately symmetrical with respect to the distribution of the central axis, indicating no publication bias in the study (Additional files 1 and 2).

**Table 6:** Publication bias test by egger’s test. A systematic review and meta-analysis on the waist height and chronic kidney disease, 1998–2019

Obesity index	Male	Female
	*P-value*	*P-value*
BMI	0.708	0.931
WC	0.679	0.129
WHtR	0.687	0.116

## Discussion

Chronic kidney disease pathology includes chronic kidney structure and dysfunction due to various causes. It is an important risk factor for early stages of end stage renal disease (ESRD) as well as the prevalence of and death due to cardiovascular disease [20]. The prevalence of CKD has increased significantly and has become an important public health issue in China [21]. Previous studies have shown that Asians are more prone to central obesity [22], and the risk of metabolic disease is highest in cases of abdominal and visceral fat distribution. Since obesity is an important risk factor for CKD, it is important to find indicators that can better reflect the level of obesity to predict the occurrence and development of CKD.

The combined results of all populations in this study showed that the highest AUC combined value is for WHtR, regardless of gender. Further, in research based on gender stratification, the combined value of male WHtR was greater than female. This is consistent with existing research results [23, 24], and suggests that WHtR can better reflect the influence of obesity on CKD than BMI, WC, and other indicators.

Aside from the AUC area of WHtR in predicting CKD is the biggest, and it still has obvious advantages in methodology application. BMI is influenced by height and weight. Those with the same height but larger degree of body muscle may have the same BMI as those who have high fat weight. Those who are tall with low WC and those who are short with large WC may also have the same BMI. Therefore, the diagnosis of obesity based on BMI alone does not reflect the effect of obesity on the risk of chronic kidney disease. Although WC can better reflect the accumulation of abdominal fat [25], the gender and ethnic distribution of WC is significantly different. At the same time, WC cannot reflect the influence of height on body fat distribution, the use of WC alone may overestimate the risk of obesity associated with tall people or underestimating short stature [26]. When body type changes, WC and hip circumference increase or decrease in proportion, so WHR cannot accurately reflect changes in abdominal fat. Also, the measurement of hip circumference is prone to produce random errors. The use of WHR is limited in large-scale epidemiological surveys.

WHtR balances the influence of height, and the height of adults is relatively stable. Compared with WC and WHR, WHtR can better reflect central obesity in different body types. The WHtR measurement method is easy to grasp and simple to calculate. It is especially suitable for large-scale population surveys and use in areas with insufficient resources. The results of this study also support the use of WHtR as a measure of the effect of obesity on CKD. The combined value of WHtR in males is greater than that in females. This may be due to the fact that the ages of the individuals included in this study were mostly middle-aged and older. In this age group, male abdominal obesity is more relevant than females [19].

Considering that the influence of obesity in different ages and ethnicities on CKD may be different in men and women, this paper analyzed four studies using Chinese populations as subjects and discusses the applicability of the combined results to Chinese population. It was found that the AUC combined value of WHtR was the largest among men and women, the combined value of WHtR in men is greater than that in women suggesting that WHtR may be a good measure of the effect of obesity on CKD in Chinese adults, especially in Chinese adult male populations. Its predictive value for Chinese adult male CKD is higher than that of Chinese women.

A large number of studies have shown that obesity is closely related to the occurrence of CKD, but the specific mechanism by which obesity interacts with CKD is not fully understood. It is currently believed that the association between obesity and CKD may be achieved through a variety of biological mechanisms, such as hormonal factors, inflammation, oxidative stress, and endothelial function [27]. One study by Tanaka et al. [28] selected 74 patients with pathologically proven IgA nephropathy and divided them into BMI subgroups. They found that the obese group had significantly increased glomerular and a thickened glomerular basement membrane (GBM), which was significantly different from the control group. This ultrastructural change in the kidney may be the main cause of obesity-induced increase in urine protein. A separate study has shown that there is a significant positive correlation between proteinuria and central obesity at baseline, persistent proteinuria is also an early sign of chronic kidney disease [29].

Adipose tissue is an active endocrine organ, and several adipokines, including leptin and adiponectin, may be involved in the pathogenesis of CKD. Excessive adipose tissue can cause activation of the sympathetic and renin angiotensin systems, as well as lipid deposition, ultrafiltration, and increased sodium reabsorption in the kidneys. This forms a feedback loop in which obesity-induced decline in renal function accelerates the progression of hypertension, and worsening of hypertension further exacerbates the impairment of renal function. Other adipose tissue-derived factors, such as tumor necrosis factor-beta, interleukin-6, and plasminogen activator inhibitor 1, may also cause damage to kidney function [30].

The diagnostic criteria for CKD in this study were eGFR< 60 mL/(min·1.73m^2^). This index was calculated using the CKD-EPI equation and the ratio of urinary albumin to urinary creatinine. Since most of the included studies are cross-sectional, it is impossible to estimate the duration of kidney dysfunction, so patients with acute kidney injury cannot be excluded. Also, because some of the literature was incomplete, it is not possible to stratify all included studies by gender. Due to the limitations of cross-sectional studies, the causal relationship between obesity and the development of CKD cannot be determined, and long-term prospective studies are needed for validation.

## Conclusions

In summary, WHtR appears to be a better measure of central obesity, relative to other indicators, and is an easy-to-use. Additionally, WHtR measurement is a noninvasive tool for identifying individuals at risk of developing obesity-related CKD to to ensure the appropriate steps (e.g., lifestyle modification) are initiated as early as possible to reduce the burden of diseases. It could be validated as a predictive indicator of CKD after being validated by high-quality prospective studies. Of course, given that obesity is modifiable risk factor, controlling weight is an important factor in preventing the development of CKD [31].

## Additional files

Additional file 1: Funnel plot of CKD predicted by WHtR of male based on gender stratification.

Additional file 2: Funnel plot of CKD predicted by WHtR without gender stratification.

## Data Availability

We declared that materials described in the manuscript, including all relevant raw data, will be freely available to any scientist wishing to use them for non-commercial purposes, without breaching participant confidentiality.

## Abbreviations

AHRQ: Agency for Healthcare Research and Quality
AUC: Area under the curve
BMI: Body mass index
CI: Confidence interval
CKD: Chronic kidney disease
eGFR: estimated glomerular filtration rate
ESRD: End-stage renal disease
GBM: Glomerular basement membrane
NOS: Newcastle Ottawa scale
WC: Waist circumference
WHR: Waist-to-hip ratio
WHtR: Waist circumference height ratio
