# Supplementary figures and images for: Waist height ratio predicts chronic kidney disease: a systematic review and meta-analysis, 1998–2019
Source: Arch Public Health. 2019 Dec 18;77:55. doi: 10.1186/s13690-019-0379-4 (PMC6918668; doi:10.1186/s13690-019-0379-4)

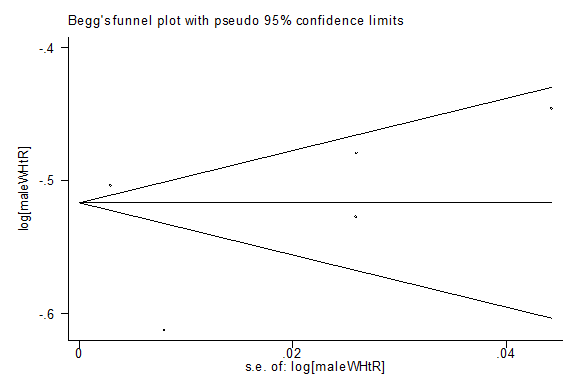

Supplement: Supplementary file 1 — Funnel plot of CKD predicted by WHtR of male based on gender stratification. [file 13690_2019_379_MOESM1_ESM.png]

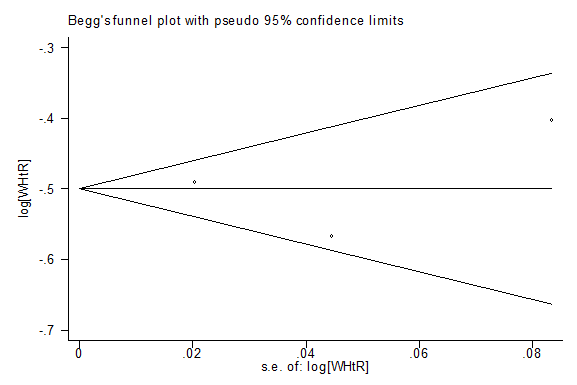

Supplement: Supplementary file 2 — Funnel plot of CKD predicted by WHtR without gender stratification. [file 13690_2019_379_MOESM2_ESM.png]
